# Priming the body to receive the therapeutic agent to redefine treatment benefit/risk profile

**DOI:** 10.1038/s41598-018-23140-9

**Published:** 2018-03-19

**Authors:** Matthieu Germain, Marie-Edith Meyre, Laurence Poul, Marion Paolini, Céline Berjaud, Francis Mpambani, Maxime Bergere, Laurent Levy, Agnès Pottier

**Affiliations:** NANOBIOTIX, 60 rue de Wattignies, Paris, 75012 France

## Abstract

Many therapeutic agents offer a low useful dose (dose responsible for efficacy)/useless dose (dose eliminated or responsible for toxicity) ratio, mainly due to the fact that therapeutic agents must ensure in one single object all the functions required to deliver the treatment, which leads to compromises in their physico-chemical design. Here we introduce the concept of priming the body to receive the treatment by uncorrelating these functions into two distinct objects sequentially administered: a nanoprimer occupying transiently the main pathway responsible for therapeutic agent limited benefit/risk ratio followed by the therapeutic agent. The concept was evaluated for different nature of therapeutic agents: For nanomedicines we designed a liposomal nanoprimer presenting preferential hepatic accumulation without sign of acute toxicity. This nanoprimer was able to increase the blood bioavailability of nanomedicine correlated with a lower hepatic accumulation. Finally this nanoprimer markedly enhanced anti-tumor efficacy of irinotecan loaded liposomes in the HT-29 tumor model when compared to the nanomedicine alone. Then, for small molecules we demonstrated the ability of a cytochrome inhibitor loaded nanoprimer to increase efficacy of docetaxel treatment. These results shown that specific nanoprimers could be designed for each family of therapeutic agents to answer to their specific needs.

## Introduction

**Figure 1:**
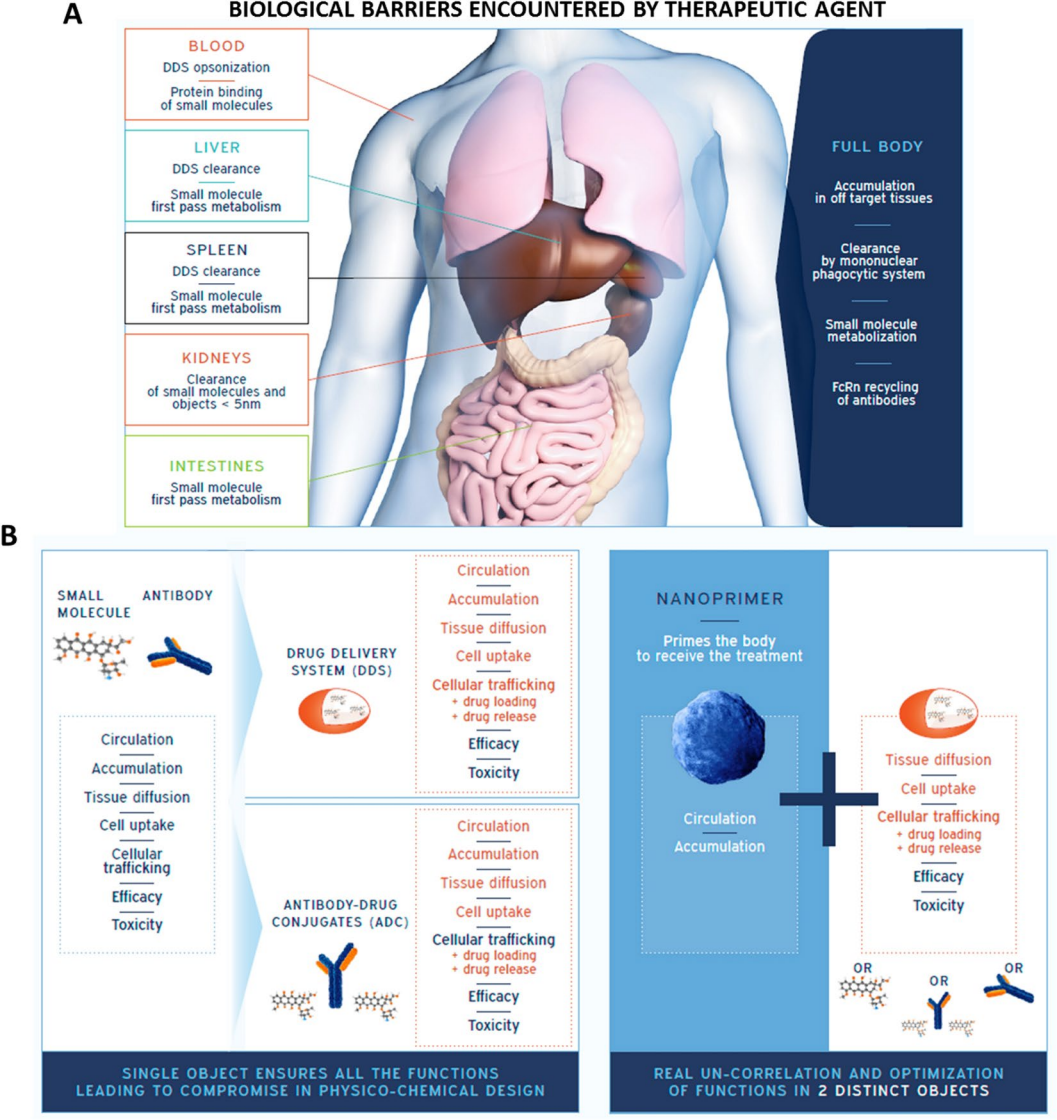
Therapeutic agent’s bioavailability: functions un-correlation for optimization: (**A**) For an optimized bioavailability a therapeutic agent must overcome clearance/metabolization mechanisms and accumulation in healthy tissues responsible for potential toxicity. Optimization of therapeutic agent bioavailability requires modification of its physico-chemical properties. (**B**) Therapeutic agents, such as small molecules and antibodies require a small size to ensure their chemical mode of action resulting in high level of compromise in their physico-chemical attributes leading to non-optimized biodistribution and poor efficacy - toxicity ratio. Antibody drug conjugate (ADC) and drug delivery system (DDS) have been developed to optimize the biodistribution of existing drugs. These approaches aim at obtaining a better efficacy - toxicity ratio but are still limited since physico-chemical attributes of the object maintain a high level of compromise. Our approach is intended to prime the body to receive the treatment by sequential administration of a nanoprimer and the therapeutic agent. The nanoprimer is designed to physically and transiently occupy organs responsible for therapeutic agent low efficacy/toxicity profile.

The benefit of a therapeutic agent is due to its bioavailability and intrinsic efficacy balanced with its toxicity profile^1–3^; namely the therapeutic agent should exhibit sufficient blood bioavailability (circulation) for efficient accumulation at the target site, and appropriate diffusion in the target tissue, with additional requirements regarding cellular uptake and subcellular localization. So far, a large part of the administered dose remains useless due to the high rate of metabolism^4–6^ and clearance. Another part of the dose could also be useless in reason of therapeutic agents accumulation in off-target tissues^7^ (Fig. 1A). Moreover, circulation and accumulation in off-target tissues are potentially associated with increased toxicity^8,9^. Hence, the cost effectiveness could be raised by improving the useful quantity of therapeutic agents compared to the actually administered dose.

Biodistribution, efficacy and toxicity profile of a therapeutic agent are driven by the physico-chemical interactions between the therapeutic agent and biological entities in the body, making the selection of its physico-chemical properties challenging to optimize treatment outcomes (Fig. 1B). Compromises in the design of physico-chemical attributes depend on the nature of the considered therapeutic agent: the chemical mode of action of small molecules at the subcellular level defines their molecular size range, which is closely related to their high probability to undergo first pass elimination through liver and intestines as well as renal clearance^10^. Biologicals such as monoclonal antibodies are, by design, long circulating agents. However, their long circulation in the blood may ultimately result in adverse toxicities in peripheral tissues^11^. Antibody Drug Conjugates (ADC) and drug delivery systems (DDS) have contributed to optimize the efficacy/toxicity profile of small molecules by modifying their biodistribution^12,13^. However, results obtained with all these recent approaches were lower than expected since the design of the object still requires compromises in terms of physicochemical properties (Fig. 1B). For example, stealth liposomes, mostly obtained by pegylation, have been shown to decrease recognition by the mononuclear phagocytic system (MPS) and lead to a prolonged circulation, but also to limit recognition and uptake by target cells required for an efficient delivery of the active principle^14,15^. Furthermore, the prolonged circulation of these liposomes may lead to an accumulation in off-target tissue, typically the skin, resulting in new toxicity^9^ without obvious increase in the efficacy of the treatment^16^.

## Could we prevent compromise?

Each therapeutic agent suffers from its own limitations. Considering several families of therapeutic agents categorized by size, namely the nanomedicine products (5–1000 nm^17^, typically 100–200 nm for DDS)^18^, the monoclonal antibody (biologicals) products (10–15 nm)^19^ and the small molecules (typically below 2 nm), specific bioavailability limitations can be identified for each family (as mentioned previously). These specific limitations can be overcome using the same global approach: redefine their biodistribution by priming the body to receive the therapeutic agent. This approach relies on the sequential administration of a first object, the nanoprimer and a second object, the therapeutic agent (Fig. 1B) to uncorrelate functions in two objects. The nanoprimer is a nanoparticle designed to transiently occupy the main pathway responsible for the useless dose, *i*.*e*. part of the therapeutic agent for which bioavailability is not optimized. As such, the nanoprimer redefines the biodistribution of the existing compound, thus increasing the useful dose without impacting its physico-chemical attributes to fully optimize its efficacy/toxicity profile (Fig. 1B). Based on this global approach, we intend to design different nanoprimers to answer specific needs of the above mentioned therapeutic agent families and obtain universal nanoprimers to be insert in treatment standard of care.

### Nanomedicine products

A recent retrospective analysis on the accumulation of nanomedicines (essentially DDS) in tumors^20^ showed that among products that have been developed in the last decade, a median accumulation of only 0.7% of the injected dose was recovered in solid tumors. Although this parameter may not reflect the overall benefit/risk ratio that nanomedicine products may bring^21^, there is still potential for improvement of this ratio.

Despite intensive researches to optimize the physico-chemical attributes of nanomedicine products, they remain highly recognized by the main organs of MPS, liver and spleen, leading to a fast and high percentage of trapping of the administered dose^22^.

The size remains one of the main driver of nanomedicines distribution and clearance by the MPS. Large objects (>100 nm) are preferentially cleared by the Kupffer cells present in hepatic sinusoids, whereas the fenestration of the Disses’ space allows particles with sizes below approximately 100 nm to reach the hepatocytes.

Other physico-chemical attributes are also responsible for the rapid and extensive trapping of nanomedicine products by the MPS. Charged particles are more prompt to be cleared by the MPS^23^ and positively charged particles may trigger toxicity^24^. Besides, chemical groups anchored on the surface of the nanoparticles, nanoparticles’ shape^18^ and hardness^25^ are also involved. Therefore, distribution and clearance of nanomedicines by the MPS is dictated by a set of physico-chemical attributes that must be considered as a whole, and cannot be optimized individually.

## “Keep liver busy” to increase useful dose/useless dose ratio

**Figure 2:**
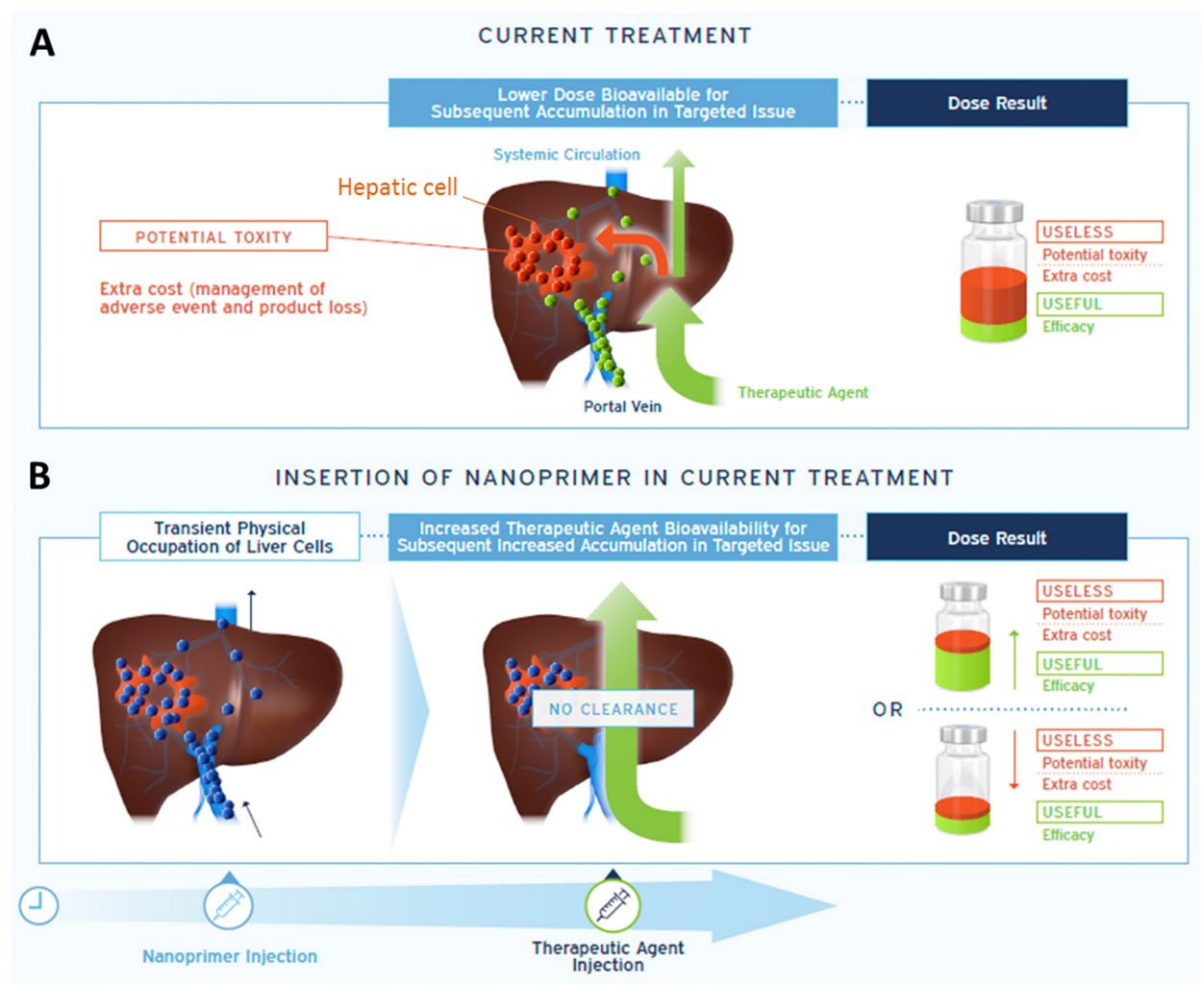
Nanoprimer: towards therapeutic agent’s bioavailability optimization via a sequential administration. The accumulation of the nanoprimer in liver decreases the clearance of the therapeutic agent to obtain an increased efficacy for a same dose administered or to decrease the toxicity (and extra-cost of adverse events management or product loss) for a same efficacy.

We propose to “keep the liver busy” by sequentially administering (i) a nanoprimer designed to physically and transiently saturate the Kupffer cells and (ii) a nanomedicine product (Fig. 2). The nanoprimer aims at priming the body to limit the capture of the nanomedicine product by the Kupffer cells. The expected outcomes are twofold: enhanced useful dose: the diminution of the nanomedicine product clearance by the MPS will enhance its blood bioavailability with an expected increase in treatment efficacy; decreased useless dose: correspondingly for a similar efficacy, the treatment toxicity could be reduced (and therefore adverse events more easily managed). Bioavailability raise using RES blockade has already been described by different approaches such as macrophage depletion with chlodronate loaded liposomes^26^ or pre-dosing of the liver with empty DDS^27^. An article from Liu *et al*.^28^ presents a correlation between liver saturation and increased treatment benefit/risk ratio using commercial empty liposomes presenting a broad size distribution (e. g. between 0,3 and 3 µm; phosphatidyl choline/cholesterol composition leading to low negative charge surface). These results are promising but it has been shown that biodistribution could be orientated by tuning physico-chemical attributes of the nanoparticles: as example size below 200 nm would increase hepatic accumulation compared to nanoparticles larger than 200 nm showing splenic accumulation, and highly negative surface charge would enhance macrophages internalization compared to neutral nanoparticles^29,30^. Our approach is focused on hepatic saturation since liver contains 80% of endogenous macrophages. This implies that the design of nanoprimer physico-chemical attributes must take into account the specificity of hepatic structures to maximize nanoprimer hepatic accumulation and interaction with Kupffer cells. Required physico-chemical attributes are listed in Table 1. Typically the size should be in the range 100–200 nm in order to avoid diffusion through Disse’s space of sinusoidal capillaries^31^ and limit splenic accumulation. Size below 200 nm to obtain a high curvature radius^32^ and appropriate surface composition^33,34^ are important to limit complement interaction. Spherical shapes have been described as more in favor of liver accumulation compared to elongated shapes^18^.

**Table 1:** Design requirement of the biocompatible nanoprimer for nanomedicine bioavailability increase: Determination of physico-chemical attributes of liposomes to be used as nanoprimer to enhance liver accumulation and interaction with Kupffer cells.

Physico-chemical attributes	Targeted values for an optimized interaction with Kupffer cells and safety	Liposome as biocompatible nanoparticle
Size *For optimized interaction*	>100 nm to limit diffusion in Disse’s spaces^31^ and <200 nm to limit proteins interaction due to lower curvature radius^32^ and spleen uptake^18^	**150 nm**
Surface properties *For optimized interaction*	Negative charge to enhance interaction with cell membrane. No positive charge to avoid toxicity and complement activation^33^	**−88 mV** Composition: -50%mol Succinyl phosphatidyl-ethanolamine -50% mol cholesterol
Shape *For optimized interaction*	Spherical nanoparticles of about 150 nm present a good accumulation in liver versus spleen or lung compared to other cylindrical or discoidal shapes^18^	**Spherical shape**
Degradability *For safety*	Highly biodegradable materials ideally in less than 24 hours	**Liposomes disrupt in few hours once in lysosomes**, followed by degradation/recycling of phospholipids and cholesterol^34^

Here we design a liposomal nanoprimer with specific physico-chemical attributes (Table 1) to enhance its liver accumulation^2^, develop direct physical interactions with Kupffer cells without unwanted biological interaction, and^3^ transiently and physically saturate Kupffer cells to redefine nanomedicines bioavailability. Furthermore, since bioavailability raise is not surely sufficient to improve treatment benefit/risk ratio (potential new toxicity, importance of other parameters such as target tissue diffusion or cell uptake), the impact of liposomal nanoprimer on a nanomedicine benefit/risk ratio should be evaluated.

**Figure 3:**
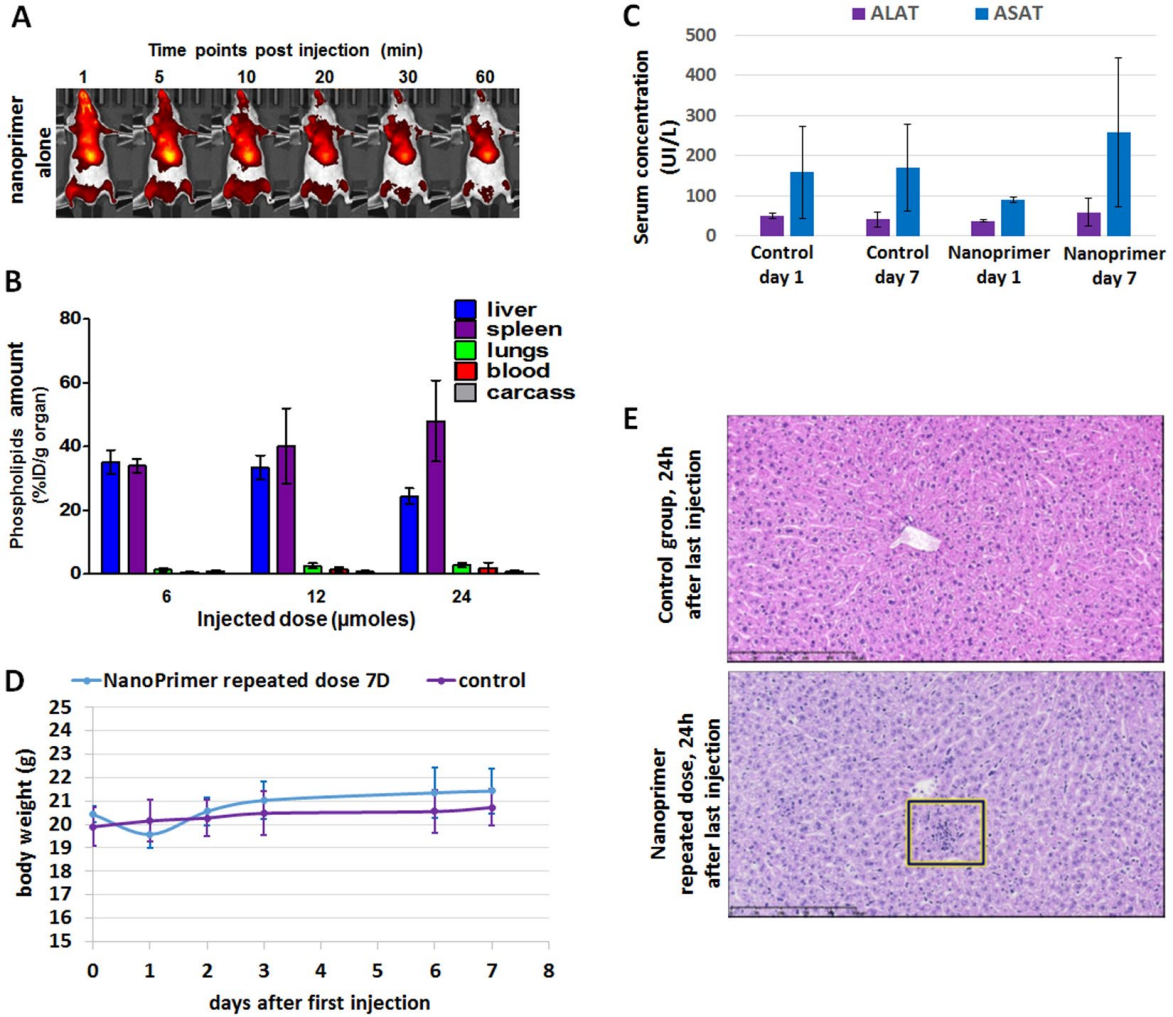
liposomal nanoprimer biodistribution and acute toxicity evaluation. (**A**) Fluorescently labelled nanoprimer was intravenously injected alone on mice. Then, fluorescence acquisitions were performed on whole mice during 1 h using *In Vivo* Imaging system (IVIS). A massive hepatic accumulation was observed in the 10 min following injection. (**B**) Dose dependent accumulation of nanoprimer in liver, spleen, lungs, blood and carcass: Accumulation was measured by encapsulation of gold nanoparticles inside liposomes and quantification of gold in the different organs by inductively coupled plasma mass spectrometry 24 h after product injection (10 mL/kg) in mice (n = 5). Phospholipids amounts per g of organ (%ID/g) were extrapolated from Au quantified in organs reported to the ratio of Au/phospholipids of the injected nanoprimer solutions. Data are mean +/− SEM. (**C**) Serum concentration of aspartate aminotransferase (ASAT) and alanine aminotransaminase (ALAT) 1 or 7 days after last intravenous injection of 3 doses of 85 mM; 10 mL/kg nanoprimer (median, n = 4). Control mice received 3 doses of Hepes/NaCl (25 mM/145 mM) (median, n = 4). (**D**) Body weight evolution of mice during the 7^th^ days after the same treatment as in (**B**) (n = 4). (**E**) Liver histological observations performed 24 h after last injection of the 3 doses of nanoprimer or Hepes/NaCl. Square defines an example of mild grade mixed inflammation areas observed 24 h after last injection of maximized dose of nanoprimer. These areas remain only visible at minimal grade on 1 of 4 mice 7 days after last injection of nanoprimer. Scale bar: 250 µm.

Figure 3A shows preferential accumulation of the liposomal nanoprimer in the liver within the 10 min after intravenous injection in mice, meaning that a time schedule of 10 min could be used between the injections of nanoprimer and nanomedicine. This result was confirmed by ICPMS titration (Fig. 3B), showing that nanoprimer was accumulated in liver, spleen and lungs of nude mice 24 hours following its administration (bolus systemic injection). The low quantity of phospholipids inferred from ICPMS titration of gold in the blood revealed the absence of prolonged circulation. ICPMS titration confirmed a preferential accumulation of the nanoprimer in the liver 24 h after injection. Minimal accumulation was observed in the lungs and other organs regrouped into carcass, whatever the tested dose (between 20 and 80 mM in lipids).

To assess potential acute toxicity of the liposomal nanoprimer we exposed mice to a maximized dose of biocompatible liposomes. Three injections of 85 mM nanoprimer solution spaced of 24 h were performed, then we assessed body weight evolution and serum levels of aspartate aminotransferase (ASAT) and alanine aminotransaminase (ALAT) 1 and 7 days after last injection. No marked difference was observed in ASAT and ALAT levels between control group (three injections of Hepes/NaCl) and treated group neither 1 day nor 7 days after last injection (Fig. 3C). Equally, no variation on other evaluated biochemical parameters (albumin, total proteins, urea, supplementary figure S1) was observed compared to control. Furthermore, there was no difference in body weight evolution between treated and control groups (Fig. 3D). Histological observations were also realized on liver and spleen (Fig. 3E). Only hepatic mild mixed inflammatory cell infiltration was observed 24 h after injection on the 4 mice of treated group but this infiltration was transient (1/4 of treated group animals still showed minimal infiltration 7 days after injection). Since infiltrations were minimal to mild in severity and not associated with ASAT/ALAT increase they were considered as not adverse and reversible. Taken together these data suggest that nanoprimer can be safely administered using a single dose of 85 mM. Further investigations will be realized to evaluate toxicity of nanoprimer combined with relevant therapeutic agent.

**Figure 4:**
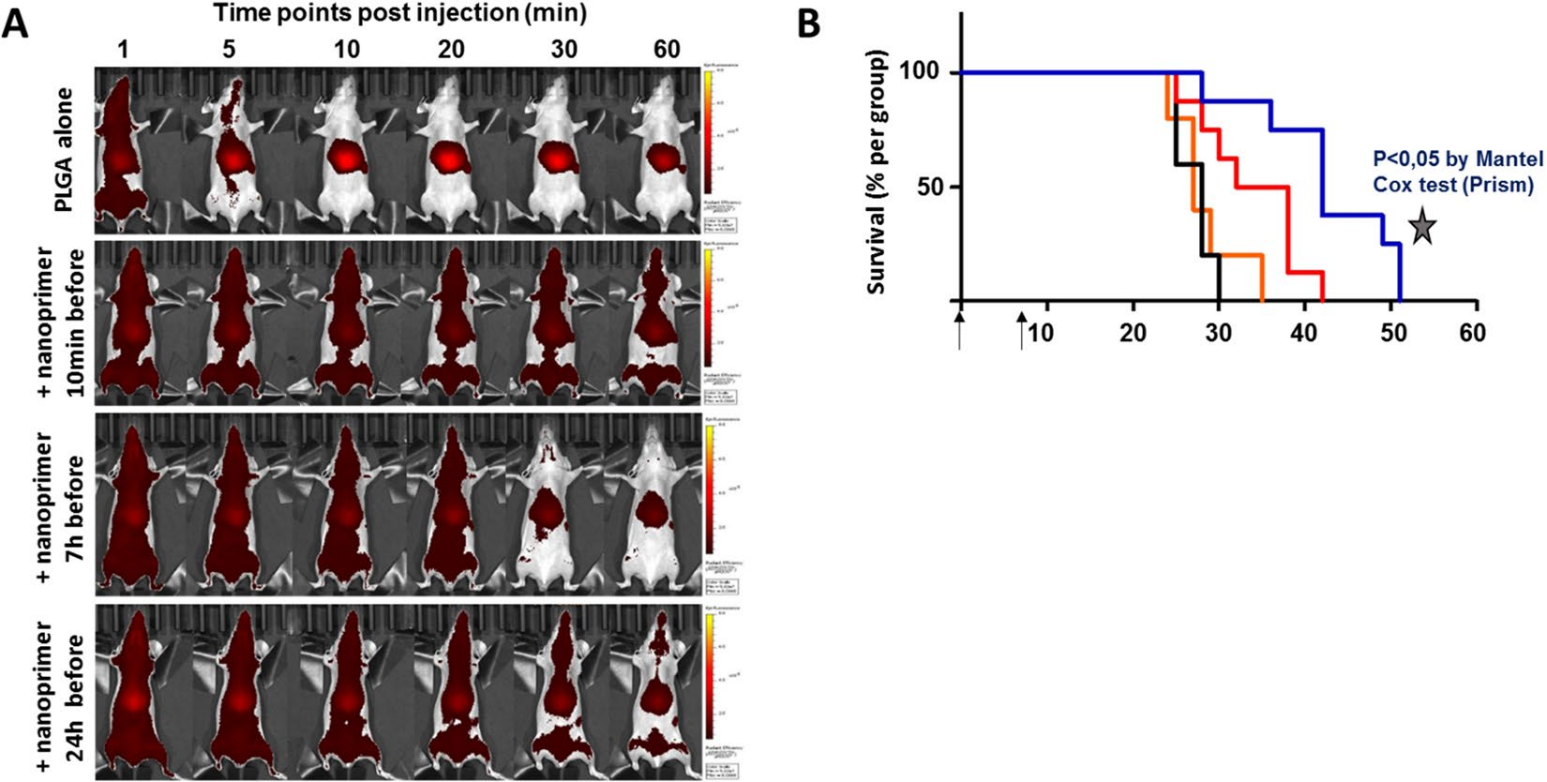
Liposomal nanoprimer impact on nanomedicines blood bioavailability and anti-tumor efficacy on HT-29 tumor model. (**A**) PLGA nanoparticles were selected as this polymer is synthetic, biodegradable and used in drug delivery systems approved by FDA (EligardR, zoladexR) or in clinical trial (Bind-014)^53,54^. PLGA nanoparticles were intravenously injected alone on mice or after intravenous injection of liposomal nanoprimer with various time schedule (10 min, 7 and 24 h). Then, immediately after PLGA nanoparticles injection, fluorescence acquisitions were performed on whole mice during 1 h using *In Vivo* Imaging system (IVIS). Increased blood bioavailability for at least 1 hour is observed for the PLGA nanomedicine-based product administered 10 min after the liposomal nanoprimers when compared to the PLGA nanomedicine-based product alone. This enhanced blood availability is correlated with a lower accumulation of the product in the liver and spleen and without noticeable accumulation in other organs. The same experiment repeated with the PLGA nanoparticles injected 7 h or 24 h after the liposome as nanoprimers demonstrates that PLGA nanoparticles blood bioavailability is still increased by nanoprimers but in a lower extend since this effect decreases after 30 min highlighting transient MPS (liver and spleen) occupancy by the nanoprimers. **(B)** Kaplan-Meier diagram of camptothecin 11 (CTP11) liposomes +/− nanoprimer: mice were xenografted with HT-29 colorectal tumor model and randomized when the mean tumor volume reached 150 mm3. Mice were treated as follow: Control Hepes/NaCl (25 mM/145 mM) (orange curve, n = 5); nanoprimer alone 85 mM (black curve, n = 5); CPT11-liposomes 15 mg/kg alone (red curve, n = 8) and with nanoprimer 85 mM injected 10 min before each CPT11-liposomes injection (blue curve; n = 8). For all groups, intravenous injections were performed at days 0 and 6 (black arrows). Survival difference between the CPT11-liposomes alone and CPT11-liposomes with nanoprimer groups was evaluated with Mantel-Cox test, * p-value < 0.05.

Then, the ability of liposomal nanoprimer to decrease nanomedicine clearance by the liver was tested with 200 nm fluorescent PLGA nanoparticles injected 10 min, 7 h or 24 h after liposomal nanoprimer, both intravenously. Figure 4A shown that administration of liposomal nanoprimer 10 min before PLGA nanoparticles leads to increase PLGA nanoparticles blood bioavailability. This increase is transient since the impact of liposomal nanoprimer progressively decreased when time between nanoprimer and PLGA nanoparticles injections was increased to 7 and 24 h.

Finally a preliminary proof of concept of increased efficacy was performed using a CPT11 (irinotecan) loaded liposomes of 200 nm size as model nanomedicine administered intravenously 10 min after the liposomal nanoprimer in HT-29 human colorectal tumor model in nude mice (Fig. 4B). Results shown a significant prolonged overall survival for nanoprimer + CPT-11 liposomes treated group as compared to group treated with CPT-11 liposomes alone. Further experiments are now mandatory to^1^: define the dose of nanoprimer required to ensure the physical hepatic saturation^2^, evalute the impact of the defined dose of nanoprimer on nanomedicine biodistribution and^3^ perform a long term toxicity of the nanoprimer alone and of the combination product (nanoprimer + nanomedicine).

### Small molecules treatment

Phase I metabolic enzymes play a key role in the fate of the majority of small molecules^35^. Most of phase I drug metabolism is handled by the cytochrome P450 (CYP450) enzymes, which due to genetic polymorphisms leads to high inter-individual variability^36,37^. This inter-patient variability leads to either drug under-dosing, with a lack of efficacy - or drug over-dosing, frequently generating side effects. Many major drugs are effective in only 50% to 75% of patients^38^ and more than 2 million cases of adverse drug reactions occur annually in the United-States^39^.

## Keep hepatocyte busy to decrease useless dose

We propose to “keep the hepatocyte busy” by sequentially administering (i) a nanoprimer which is designed to transiently inhibit the CYP 450 in hepatocytes and (ii) a small molecule. The nanoparticles aim at priming the body to receive the treatment by inhibiting the drug’s hepatic metabolism.

Nanocarriers designed to target hepatocytes and encapsulating natural compounds with known metabolism-blocking capacity are interesting objects to be used as nanoprimers. The expected outcomes are the following:

- For a given efficacy, decreasing of the useless dose and of the potential associated toxicity (small molecule hepatotoxicity and/or metabolites toxicity) by increasing the drug bioavailability when compared to the drug alone,
- Normalizing the drug dosing by reducing high inter-individual variability (*i*. *e*. normalization of small molecules metabolization by hepatic CYPs).

**Figure 5:**
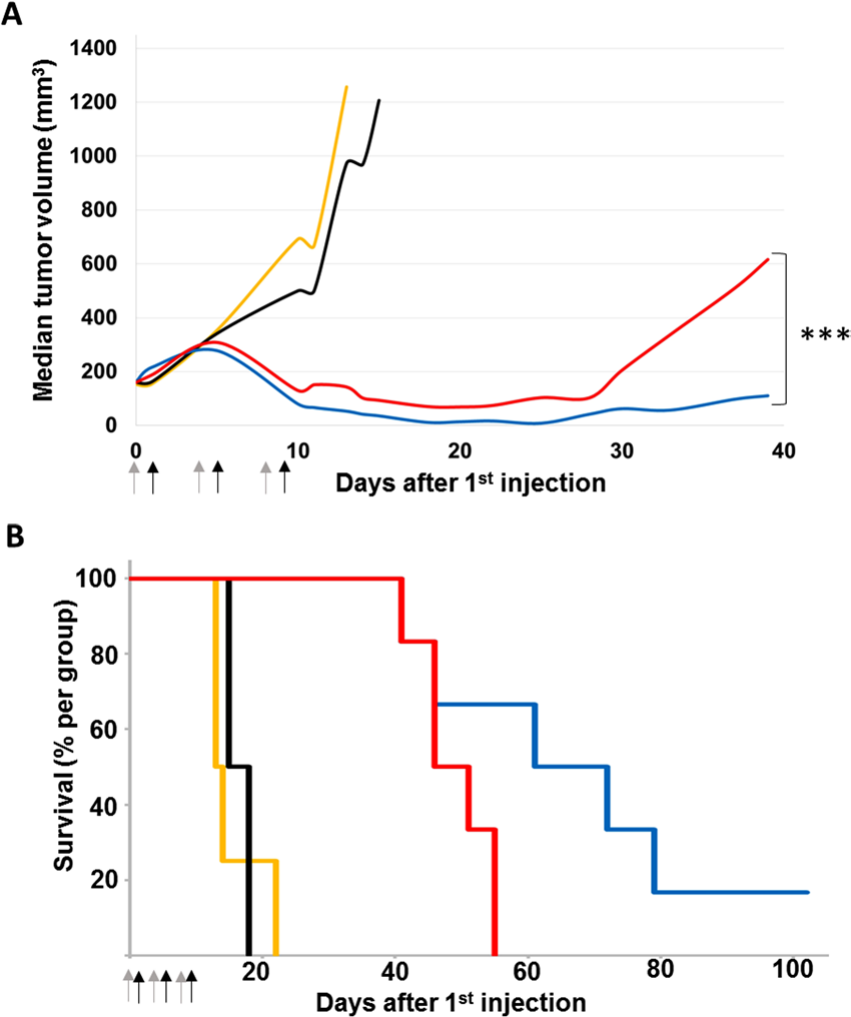
Impact of bergamottin loaded PLGA nanoparticles functionalized with galactosamine (BM-PLGA-Ga) as nanoprimer on antitumor efficacy on MDA-MB-231 tumor. Mice were xenografted with MDA-MB-231 breast tumor model and randomized when the mean tumor volume reached 200 mm^3^. Mice were treated as follow: control NaCl 0.9% IV injected on days 0, 4 and 8 (orange curve; n = 4); control BM-PLGA-Ga nanoparticles alone (BM = 97 µg/kg) IV injected on days 0, 4, and 8 (black curve; n = 4); Docetaxel 20 mg/kg IV injected with glucose 5% on days 0, 4 and 8 and docetaxel on days 1, 5, 9 (red curve; n = 6); BM-PLGA-Ga nanoparticles (BM = 97 µg/kg) IV injected on days 0, 4, and 8 and docetaxel 20 mg/kg IV injected on day 1, 5 and 9 (blue curve; n = 6). All solutions were injected at 10 mL/kg. (**A**) Tumor growth expressed as median tumor volume. Difference between the BM-PLGA-Ga/docetaxel and docetaxel alone groups was evaluated with a 2-way ANOVA analysis and a Bonferroni post-test, *** p-value < 0.001. (**B**) Kaplan-Meier diagram of overall survival. The median survival was 66 days for PLGA-Ga BM treated group, compared to 48 days for the docetaxel alone group and the overall survival rate was 67% versus 0% at day 55. Arrows: injections (grey arrows: BM-PLGA-Ga nanoparticles injections and black arrows: docetaxel injections) (Reproduced with author’s permission from Nano-sized cytochrome P450 3A4 inhibitors to block hepatic metabolism of docetaxel. Paolini, M. *et al*. International Journal of Nanomedicine. Volume 2017:12 Pages 5537–5556. DOI: https://doi.org/10.2147/IJN.S141145).

In a previous paper published by our group^40^, a preliminary proof of concept was shown using docetaxel as model drug and bergamottin (BM), a furanocoumarin compound found in grapefruit, as CYP 450 inhibitor. BM was encapsulated in a 63 nm PLGA nanoparticles functionalized with galactosamine (BM-PLGA-Ga) to enhance recognition by hepatocytes. The results were encouraging in terms of efficacy and prolonged overall survival in MDA-MB-231 breast human tumor model in nude mice, with good tolerance to treatment. Difference in tumor growth delay between the two groups, docetaxel alone and BM-PLGA-Ga + docetaxel, was statistically significant (Fig. 5A, reproduced with author’s permission from Paolini, M. *et al*. International Journal of Nanomedicine, V2017:12; 5537–5556. DOI: https://doi.org/10.2147/IJN.S141145). The median survival was 66 days for BM-PLGA-Ga treated group, compared to 48 days for the docetaxel alone group (Fig. 5B). Of note, BM-PLGA-Ga had no impact on tumor growth when compared to untreated animals (data not showed).

### Monoclonal antibodies treatment

MAbs are highly potent therapeutic agents with prolonged blood circulation. This high bioavailability is due to their size and protein structure which drive specific interactions with the neonatal Fc receptor (FcRn), preventing mAbs from lysosomal degradation and favoring their release in extracellular medium^41,42^. MAbs pharmacokinetics remain complex and subject to inter-patient variability depending on interplay of multiple factors (age, sex, body size, ethnicity…)^43,44^. Prolonged blood bioavailability of mAb allows greater tumor accumulation, but may result in increased off-target toxicity (*e*. *g*. proteinuria, bleeding, hypertension, and thrombosis observed with bevacizumab^45^. Accelerating mAb clearance would be interesting for adverse events management to prevent or limit a treatment discontinuation (e. g. proteinuria or hypertension due to bevacizumab^45^ Article from Sing Jaggi *et al*.^11^ shows that using high-dose of polyclonal IgG therapy it is possible to effectively control the blood half-lives and therefore the therapeutic index of targeted IgG antibodies via pharmacological modulation of their interaction with the protective FcRn. Such approach would be really very useful for treatment such as Bevacizumab by decreasing its concentration in blood and thus limiting its anti-angiogenic activity on healthy tissues. Its use could also decrease associated adverse events such as bleeding while preserving its anti-angiogenic activity within the target tissues. Accelerate blood clearance of IgG based on administration of intravenous immunoglobulin (IVIG) is also suitable for humoral autoimmune diseases treatment such as arthritis. Mechanism of action of IVIG is based on a saturation of the FcRn to induce accelerated clearance of pathogenic endogenous IgG involved in arthritis^46^. Moreover, it may also be suitable during co-medication to reduce potential drug-drug interactions (DDI) modifying pharmacokinetics and systemic exposure of the co-administered drug^47^. For instance, Tocilizumab targeting the interleukine-6 (IL-6) receptor decreases IL-6 signaling pathway, which contributes to reduce the activity of several CYPs. Thus tocilizumab, by inhibiting IL-6 pathway, may be responsible for higher level of CYP’s substrate metabolization that could last several weeks due to tocilizumab prolonged half-life^48^.

## Keep FcRn recycling busy to decrease toxicity

We propose to “keep the FcRn receptor busy” by sequentially administering (i) a mAb and (ii) a nanoprimer designed to transiently occupy the FcRn receptor. The nanoparticles aim at priming the body to receive the treatment by accelerating the mAb clearance.

Both mAbs and albumin are recycled by cellular FcRn recycling mechanism using their specific interaction domain on FcRn. A possible approach to decrease mAbs recycling is to design an albumin nanoparticle able to interact with the albumin domain of FcRn creating a transient steric hindrance to prevent mAbs interaction with their binding domain on FcRn. In this approach, the injection of albumin nanoparticles performed after the mAb injection may increase mAb lysosomal degradation and reduce its prolonged exposition to healthy tissues as well as decreasing DDI in the context of co-medication.

### Perspectives

Improving the efficacy/toxicity profile of therapeutic agents remains challenging as their current design involves compromises in their physico-chemical attributes and as the slightest modification of one property impacts the biodistribution, efficacy and toxicity profile of the compound. We propose a new approach focused on priming the body to receive these therapeutic agents. This approach relies on the transient occupation of the main pathways involved in the limitation of efficacy/toxicity profile of the therapeutic agent. Considering the delivery of therapeutic agents following systemic injection, the transient occupation by a nanoprimer of Kupffer cells, CYP 450 or FcRn receptors may benefit to a wide variety of existing products by modulating their bioavailability. A specific nanoprimers could be designed for each family of therapeutic agents and be inserted in the standard of care of existing treatment. Furthermore, this approach may open bright perspectives to design new therapeutic agents by shifting the notion of compromise between bioavailability, efficacy and toxicity.

## Materials and Methods

### Materials

1,2-dipalmitoyl-sn-glycero-3-phosphoethanolamine-N-(succinyl) (sodium salt) (SPE), cholesterol, 1,2-distearoyl-sn-glycero-3-phosphocholine (DSPC), 1,2-distearoyl-sn-glycero-3-phosphoethanolamine-N-[methoxy(polyethylene glycol)-2000] (ammonium salt) (DSPE-PEG) were purchased from Avanti polar lipids. Acetone, absolute ethanol (99.5%, extra dry, AcroSeal®), N-acetylgalactosamine and galactosamine, 1-ethyl-3-(3-dimethlyaminopropyl) carbodiimide (EDC) and N-Hydroxysulfosuccinimide (NHS) were all purchased from Acros Organics. Docetaxel was purchased from Accord (London, UK). 20 nm fluorescent polystyrene nanoparticles were purchased from ThermoFischer Scientific (USA). 200 nm PLGA fluorescent nanoparticles were purchased from Degradex (USA). CPT-11 and all other reagents were purchased from Sigma Aldrich unless otherwise specified.

### Methods

#### Liposomes synthesis

Liposomal nanoprimers were composed of SPE and Chol (50:50, molar ratio). They were synthetized using the thin-film hydration technique^49^. Briefly, a solution of lipids was prepared in chloroform. Chloroform was evaporated under a nitrogen stream. Then lipids film was hydrated using a Hepes 25 mM/NaCl 145 mM buffer pH 7,4. The liposomal suspension was extruded at 60 °C using gas-pressure thermostated barrel extruder (Lipex Biomembranes, Vancouver, British Columbia, Canada) through 0.2 μm polycarbonate filters (Whatman Nucleopore, Clifton, NJ) yielding a final diameter of 150 nm (%V) and a polydispersity index of 0,109 as determined by dynamic light scattering using a Malvern nanoZS. Using the nanoZS, surface charge was measured at −75mV in 1 mM NaCl; pH 7,4. The concentration of phospholipid was determined by colorimetric assay^50^.

CPT-11 loaded liposomes synthesis was performed as described in D. C. Drummond *et al*.^51^. First a solution of triethylammonium salts of sucrose octasulfate (TEA_8_SOS) was prepared by ion-exchange chromatography on a Dowex 50Wx8–200 resin in the H + form, immediately followed by titration with neat triethylamine. The TEA concentration was calculated from the amount of added TEA and was adjusted to 0.65 mol/L for TEA_8_SOS solution. The final pH of the solution was 5.5 to 6.0. Liposomes composed of DSPC/Chol/DSPE-PEG (59,7/40/0,3 molar ratio) were prepared by thin film hydration technique using theTEA_8_SOS as hydrating solution. Liposomes were formed by extrusion at 60 °C through polycarbonate membranes having a pore size of 100 nm (5 times) and 80 nm (10 times) and yielding a final diameter of 120 nm (%V) and a polydispersity index of 0,058 as determined by dynamic light scattering using a Malvern nanoZS. Unencapsulated TEA_8_SO was then removed by size exclusion chromatography on a Sepharose CL-4B column. CPT-11 (115 mg/ml, DMSO) was loaded into TEA_8_SOS-containing liposomes, with final drug-to-lipid ratio of 300 g CPT-11/mol phospholipid with incubation of the drug-liposome mixture at 60 °C (pH 6.0) for 45 minutes followed by cooling on ice for 15 minutes. Unencapsulated CPT-11 was removed by size exclusion chromatography through a Sephadex G75 column. CPT-11 concentration was determined spectrophotometrically at 372 nm in acid/methanol (20 volume % 0.5 mol/L phosphoric acid/80 volume % methanol) and phospholipids concentration by colorimetric assay as for liposomal nanoprimer.

#### Nanoprimer biodistribution study

All *in vivo* manipulations were performed on adult female mice (NMRI-Fox1nu/Foxn1nu) (Janvier, France) at the Ecole Nationale Veterinaire d’Alfort (Maisons-Alfort, France) except PLGA nanoparticles bioavailability study performed at BIOVIVO – Institut Claude Bourgelat (Marcy l’Etoile- France), according to their ethic committees policy for both, following approval respectively by ethic committees Anses/ENVA/UPEC (agreement N°14/03/17–1) and VetAgro-Sup/Lyon National Veterinary School (agreement N°1616). *In vivo* experiments were performed in accordance with relevant guidelines and regulations. Animals received good care and humane treatments.

For IVIS biodistribution study, liposomal nanoprimer were loaded with 20 nm fluorescent polystyrene nanoparticles. This liposomal nanoprimer was synthetized as described above but including fluorescent polystyrene nanoparticles in the hydration medium. After extrusion process, non-encapsulated polystyrene nanoparticles were removed from loaded liposomal nanoprimer by size exclusion chromatography on a sephacryl S-1000 column. For IVIS follow up, fluorescently labelled nanoprimer injections (20 mM; 10 mL/kg) were performed in the tail vein of anesthetized mice (Isoflurane (1–5%), oxygen (1–2 L/min)). Then animals were immediately placed in dorsal recumbency in optical imaging system IVIS Spectrum of Perkin Elmer to perform 2D fluorescent acquisitions (ex: 745 nm; em: 820 nm) starting 1 min after nanoprimer injection, every minute during 1 h on whole body. The fluorescence acquisitions were analyzed with the software Living Image version 4.4.

For ICPMS biodistribution study, liposomal nanoprimer was loaded with 12 nm gold nanoparticles (GNP). GNP were synthetized using citrate reduction approach as described in publication from Li *et al*.^52^ 2 mL of HAuCl4 (25 mM) were mixed with 8.8 mL of NaOH (20 mM), followed by adding 9.2 mL of distilled water. Then the solution was heated until 85 °C and 0.6 mL of citrate sodium (50 mg/mL) was rapidly introduced under vigorous stirring. After 1 min heating was stopped and solution was let to cool down to room temperature. GNP concentration was determined using UV-visible spectrophotometry and size by transmission electronic microscopy (data not shown). To increase their stability in various buffers, gold nanoparticles were coated with a 800 Da PEG-thiol. For this, PEG-Thiol was added in gold nanoparticles solution at pH 7,4 using a ratio of 5 molecules of Peg-Thiol per nm² of GNP surface. Solution was stirred during 3 h. Finally, GNP were reconcentrated up to 10 g/L on a polyethersulfone membrane filter of 10 kDa under a nitrogen flow. GNP were encapsulated in liposomal nanoprimer by adding GNP in the hydration buffer. Non-encapsulated GNP were removed from gold loaded liposomal nanoprimer by size exclusion chromatography on a sephacryl S-1000 column.

For biodistribution study, mice were injected in the tail vein with various concentration of gold loaded liposomal nanoprimer solutions at 10 mL/kg. 24 h after injection animals were sacrificed, organs were sampled for ICPMS titration. For these organs, homogenates were prepared at 0.1 g/mL in PBS with an Ultra – Turax, then 100 µL of homogenates was dissolved in a nitric acid (200 µL)/hydrochloric acid (600 µL) solution heated at 90 °C; 15 h. Then, 3 mL of Indium solution (5 ng/mL) and 4 mL of a 0.1% Triton X-100/1% HNO3/0.5% HCl solution were successively added at 20 °C. Solutions were vortex mixed and centrifuged 10 min at 4000 rpm before injection in an Agilent 7700× Inductively Coupled Plasma Mass Spectrometry (ICPMS) using an Agilent ASX-520 + ISIS integrated autosampler and MassHunter B01.01 acquisition software. Gold concentrations were calculated in ng/mL using standard Au nanoparticles calibration curves.

### Liposomal nanoprimer toxicity evaluation

For evaluation of toxicity mice were randomized in 2 groups of 8 animals. One group received 3 injections spaced of 24 h of 85 mM liposomal nanoprimer solution at 10 mL/kg. Control group received 3 injections of Hepes/NaCl vehicle at 10 mL/kg. For each group, 4 mice were sacrificed 24 h after the last injection and 4 mice 7 days after last injection. The following clinical biochemistry parameters were determined on blood samples: Urea, Creatinine, Total Protein, Albumin, Alanine aminotransferase (ALT), Aspartate aminotransferase (AST). Analyses were performed in Vebio facilities using a KONELAB 60 clinical chemistry analyzer through spectrophotometric methods (substrate and enzymes) and kits from Thermo scientific. For histological observations, each individual collected tissues sampled was immediately fixed in FineFix (Milestone, Bergamo, Italy), paraffin-embedded, and representative 4 to 5-μm thick sections were stained with hematoxylin and eosin (H&E). Whole slide digital scans were produced by the Hamamatsu Nanozoomer at 20× magnification. Each H&E section was thoroughly examined histologically, and lesions observed were recorded in an Excel spreadsheet, their severity graded (minimal, mild, moderate, or severe). Their distribution was also characterized (focal, multifocal, focally extensive or diffuse), as well as, their localization.

### CPT-11 loaded liposomes antitumor efficacy study

Mice were xenografted with HT-29 cells. 5 million cells in 50 μL were injected subcutaneously in the lower right flank. Tumor volume (mm3) was measured with a digital caliper and calculated with the formula: tumor volume = length*width2/2. Mice were randomized on the day of experiment, when the mean tumor volume was equal to 160 mm^3^ (S.D. 68 mm^3^). Groups were treated as shown in Supplementary Table S1. Mice were followed up for clinical signs, body weight and tumor size at least twice a week.

### PLGA nanoparticle bioavailability study

Mice were injected in the tail vein with the 85 mM liposomal nanoprimer solution at 10 mL/kg. Then injection of fluorescent PLGA nanoparticles (1 g/L; 7,5 mL/kg) were performed in the tail vein at various time schedule (10 min; 7 or 24 h) after liposomal nanoprimer injection on anesthetized animal (Isoflurane (1–5%), oxygen (1–2 L/min)), then animals were immediately placed in dorsal recumbency in optical imaging system IVIS Spectrum of Perkin Elmer to perform 2D fluorescent acquisitions (ex: 745 nm; em: 820 nm) starting 1 min after last injection, every minute during 1 h on whole body. The fluorescence acquisitions were analyzed with the software Living Image version 4.4. Animals injected with PLGA nanoparticles alone are anesthetized and immediately injected with PLGA nanoparticles.

### Bergamottin-loaded PLGA (PLGA BM) nanoparticles synthesis for docetaxel anti-tumor efficacy study

Blank nanoparticles were obtained by a modified solvent diffusion (nanoprecipitation) technique.16 Briefly, PLGA (10 mg) was solubilized in acetone (850 μL), and then 150 μL ethanol was added. This organic phase was quickly poured into 10 mL deionized water (aqueous phase) kept stirring at 1000 rpm for 3 h. Bergamottin loaded nanoparticles were prepared using the same procedure, bergamottin was added in ethanol to the organic phase. After one night at 4 °C, the non-encapsulated bergamottin was removed by filtration through 0.22-μm PES membrane. Galactosamine coatings were performed via carbodiimide chemistry, using the EDC/NHS crosslinking procedure. Typically, to 40 mL of 0.6 g/L PLGA nanoparticles suspension at pH 5, aqueous solutions containing 10.6 mg of EDC and 15.9 mg of NHS were added. After 30 minutes of incubation at room temperature, the pH was raised to 7.4 and an aqueous solution containing 2.97 mg of galactosamine was added. After sterilization by another 0.22 µm filtration, when needed, the so obtained were concentrated by 3 to 10 times, using 10 kDa PES VIVASPIN® ultrafiltration units under 192 g centrifugation. Titration of bergamottin in samples were performed by UV absorbance at 310 nm in water/ethanol (50:50, v/v), compared to the UV absorbance of a solution at the same BM concentration introduced in water/ethanol (50:50, v/v). Titration of lactic acids (D and L) was performed with colorimetric assay kits (Sigma Aldrich) according to the manufacturer’s instructions. For *in vivo* injections, glucose was added to the nanoparticles suspension (5% v/v final).

### Docetaxel antitumor efficacy studies

Mice were xenografted with MDA-MB-231 cells: respectively 5 million cells in 50 µL were injected subcutaneously in the lower right flank. Tumor volume (mm3) was measured with a digital caliper and calculated with the formula: tumor volume = length*width²/2. Mice were randomized on the day of experiment, when the mean tumor volume was equal to 170 mm3 (s.d. 34%): 4 animals per group in the control groups and 6 animals per group in the treated groups. Groups were treated as described in Fig. 4. Polysorbate-based one vial formulation docetaxel was diluted in NaCl 1% (1:9 v/v) prior to injection. Mice were followed up for clinical signs, body weight and tumor size at least twice a week. The treatment efficacy was determined using the optimal percent treated versus control ratio (%T/C), corresponding to the ratio of the mean tumor volume of treated groups versus control group and a Kaplan-Meier survival diagram.

### Data analysis

Data were analyzed and plotted on Excel (Microsoft 2013). Analysis for *in vivo* studies was performed with GraphPad Prism 5 (GraphPad Software, La Jolla, CA): statistical analyses were obtained as described in the different figures.

### Data availability statement

The datasets generated during and/or analysed during the current study are available from the corresponding author on reasonable request.

## Electronic supplementary material

supplementary information
