# Supplementary material for: Priming the body to receive the therapeutic agent to redefine treatment benefit/risk profile
Source: Sci Rep. 2018 Mar 19;8:4797. doi: 10.1038/s41598-018-23140-9 (PMC5859131; doi:10.1038/s41598-018-23140-9)
Supplement: Supplementary file 1 — supplementary information [file 41598_2018_23140_MOESM1_ESM.doc]

**Priming the body to receive the therapeutic agent to redefine treatment benefit/risk profile**

**Authors**: Matthieu Germain^1^, Marie-Edith Meyre^1^, Laurence Poul^1^, Marion Paolini^1^, Céline Berjaud^1^, Francis Mpambani^1^, Maxime Bergere^1^, Laurent Levy^1^, Agnès Pottier^1^.

**Supplementary information:**


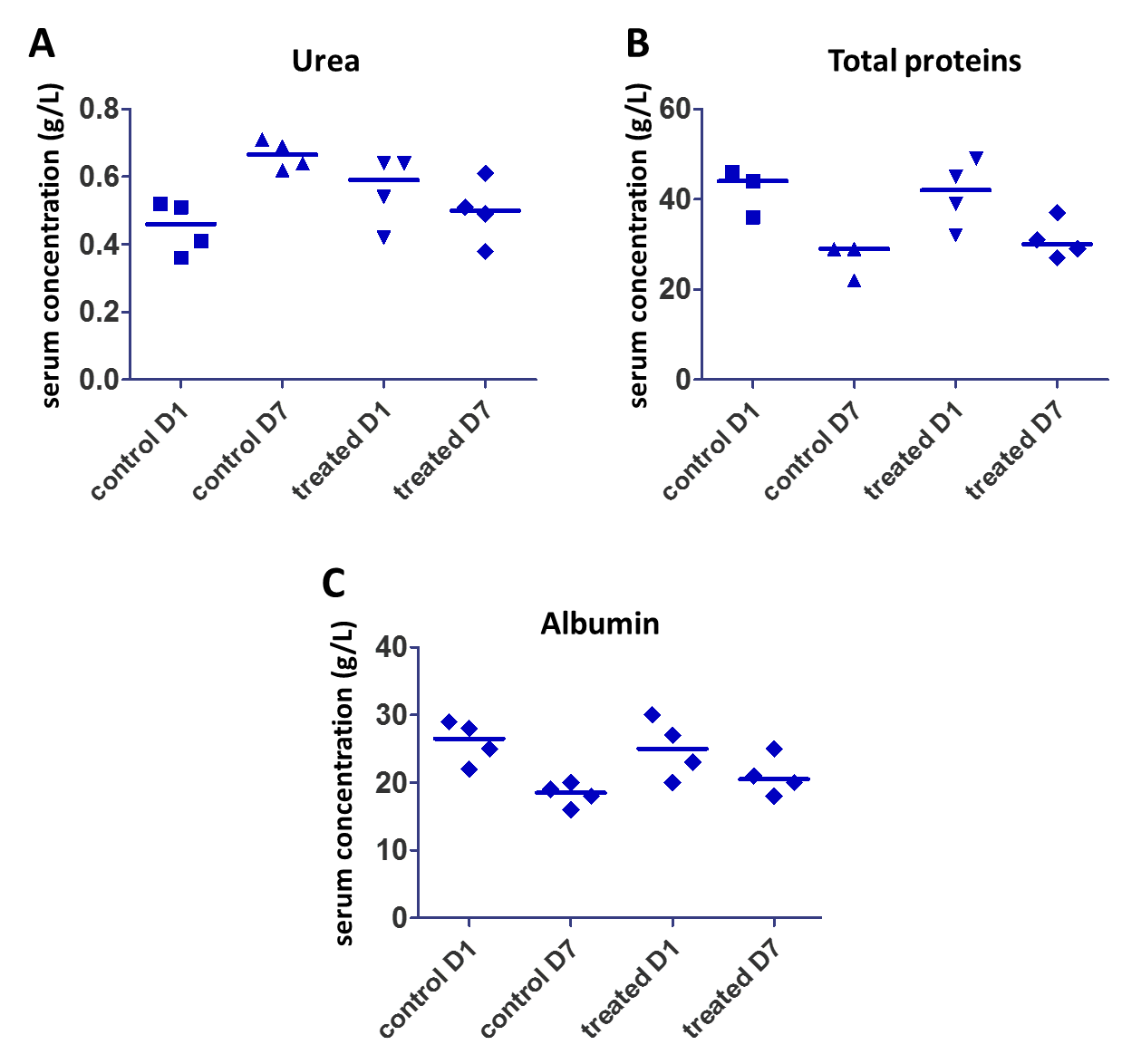


**Figure S1. Impact of nanoprimer on liver function:** Serum concentration of (A) urea (B) total proteins and (C) albumin 1 or 7 days after last intravenous injection of 3 doses of 85mM; 10mL/kg nanoprimer (median, n=4). Control mice received 3 doses of Hepes/ NaCl (25mM/145mM) (median, n=4).

| **group** | **number of mice** | **treatment** | **D0** | | **D6** | |
| --- | --- | --- | --- | --- | --- | --- |
|  |  |  | **t0** | **t0 + 10 min** | **t0** | **t0 + 10 min** |
|  |  |  |  |  |  |  |
| control | 5 | Hepes/ NaCl | Hepes / NaCl |  | Hepes / NaCl |  |
|  |  |  | 105 µL |  | 105 µL |  |
| liposomal nanoprimer alone | 5 | liposomal nanoprimer (85mM) | liposomal nanoprimer | Hepes / NaCl | liposomal nanoprimer | Hepes / NaCl |
|  |  |  | 10mL/kg | 105 µL | 10mL/kg | 105 µL |
| onivyde alone | 8 | onivyde (15 mg/kg) | ONIVYDE |  | ONIVYDE |  |
|  |  |  | 105 µL |  | 105 µL |  |
| nanoprimer + Onivyde | 8 | liposomal nanoprimer (85mM) + onivyde (15 mg/kg) | liposomal nanoprimer | ONIVYDE | liposomal nanoprimer | ONIVYDE |
|  |  |  | 10mL/kg | 105 µL | 10mL/kg | 105 µL |

**Table S1: groups for antitumor efficacy study.**
